# EpiDiverse Toolkit: a pipeline suite for the analysis of bisulfite sequencing data in ecological plant epigenetics

**DOI:** 10.1093/nargab/lqab106

**Published:** 2021-11-12

**Authors:** Adam Nunn, Sultan Nilay Can, Christian Otto, Mario Fasold, Bárbara Díez Rodríguez, Noé Fernández-Pozo, Stefan A Rensing, Peter F Stadler, David Langenberger

**Affiliations:** ecSeq Bioinformatics GmbH, Leipzig 04103, Germany; Institute for Computer Science, University of Leipzig, Leipzig 04107, Germany; Deparment of Biology, University of Marburg, Marburg 35043, Germany; ecSeq Bioinformatics GmbH, Leipzig 04103, Germany; ecSeq Bioinformatics GmbH, Leipzig 04103, Germany; Deparment of Biology, University of Marburg, Marburg 35043, Germany; Deparment of Biology, University of Marburg, Marburg 35043, Germany; Institute for Mediterranean and Subtropical Horticulture, Málaga 29010, Spain; Deparment of Biology, University of Marburg, Marburg 35043, Germany; Center for Biological Signaling Studies (BIOSS), University of Freiburg, Freiburg 79104, Germany; Center for Synthetic Microbiology (SYNMIKRO), University of Marburg, Marburg 35043, Germany; Institute for Computer Science, University of Leipzig, Leipzig 04107, Germany; Interdisciplinary Center for Bioinformatics; German Center for Integrative Biodiversity Research (iDiv) Halle-Jena-Leipzig; Competence Center for Scalable Data Services and Solutions; Leipzig Research Center for Civilization Diseases; and Leipzig Research Center for Civilization Diseases (LIFE), University of Leipzig, Leipzig 04109, Germany; Max Planck Institute for Mathematics in the Sciences, Leipzig 04103, Germany; Institute for Theoretical Chemistry, University of Vienna, Vienna 1090, Austria; Facultad de Ciencias, Universidad National de Colombia, Sede Bogotá, Colombia; Santa Fe Institute, Santa Fe, USA; ecSeq Bioinformatics GmbH, Leipzig 04103, Germany

## Abstract

The expanding scope and scale of next generation sequencing experiments in ecological plant epigenetics brings new challenges for computational analysis. Existing tools built for model data may not address the needs of users looking to apply these techniques to non-model species, particularly on a population or community level. Here we present a toolkit suitable for plant ecologists working with whole genome bisulfite sequencing; it includes pipelines for mapping, the calling of methylation values and differential methylation between groups, epigenome-wide association studies, and a novel implementation for both variant calling and discriminating between genetic and epigenetic variation.

## INTRODUCTION

Model organisms such as *Arabidopsis thaliana* have helped lay the foundation for our understanding of plant epigenetics (1–3), often proceeding DNA methylation profiling techniques such as whole genome bisulfite sequencing (WGBS) to study the DNA methylome at a nucleotide-level resolution. Historically, this practice has been considered by many as the ‘gold-standard’ for DNA methylation analysis, but can also be prohibitively expensive beyond a focus on model species (4). Cost-effective alternatives, such as affinity-based enrichment (e.g. MeDIP-seq, MDB-seq) or restriction-enzyme digestion (e.g. RRBS, MSCC), necessitate narrower hypotheses and risk spurious findings by neglecting the broader relationships detectable by more comprehensive methods. Now, the increasingly competitive costs of next generation sequencing (NGS) have opened the door for plant ecologists to apply previous lessons from WGBS on the population and community level, to gain more specific insight into non-model species (5). The EpiDiverse Toolkit addresses the challenges of expanding scope and scale for existing computational techniques, with a suite of pipelines to streamline the analysis of DNA methylation from bisulfite sequencing (bs-seq; methylC-seq) data under FAIR principles (Findability, Accessibility, Interoperability and Reusability). The aim is to provide a flexible and standardised approach when implementing ‘gold-standard’ DNA methylation analyses for non-model species in plant ecology, which additionally offers some minor improvements to further cut cost and improve computational efficiency.

The basis of bisulfite sequencing is to differentiate methylated and unmethylated cytosine nucleotides. During NGS library preparation, sodium bisulfite treatment facilitates the conversion of unmethylated cytosine to uracil while leaving 5-methylcytosine (5mC) positions intact (6). This necessitates specialised or adapted tools to carry out conventional downstream procedures such as mapping (7) and variant calling (8). For non-model plant species this is further confounded by poor quality reference genomes, with additional difficulties due in part to a high tolerance for polyploidy and high rates of heterozygosity (9). All of these aspects present difficulties in terms of running time and the optimisation of computational resources. Finally, DNA methylation can occur in additional sequence contexts (CHG, CHH) which in contrast to CG are not prevalent in mammalian data (10).

**Figure 1.**
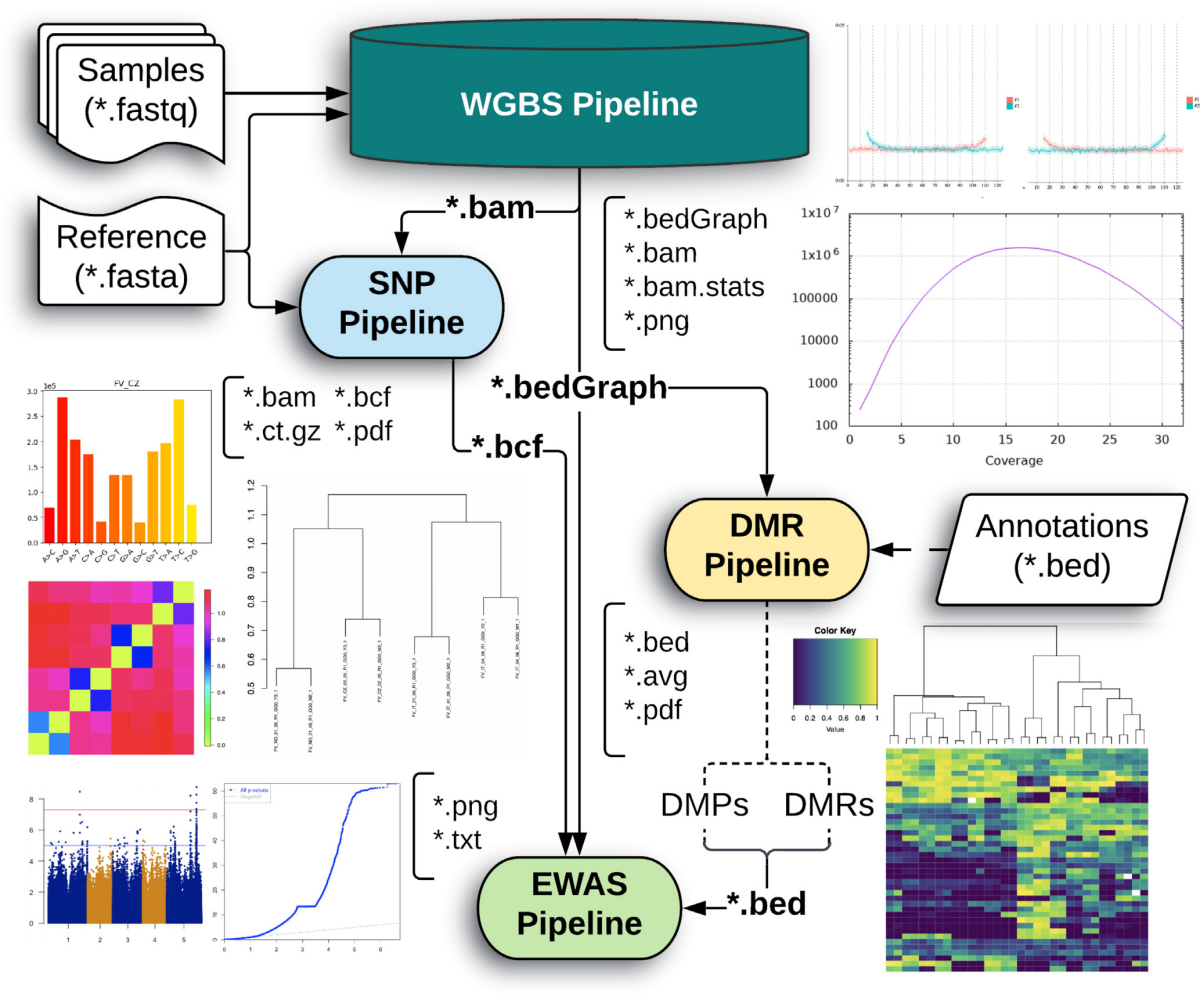
Overview of the EpiDiverse Toolkit. The WGBS data forms the foundation of the analysis, and each downstream pipeline is built to work either in cooperation with one another or, optionally, with independently-generated input data. All pipelines output runtime metadata, tracing and further visualisation in addition to what is shown here. The full output is described for each pipeline in the documentation on Github.

The tools presented herein (Figure 1) are implemented with Nextflow (11), building on best-practice concepts outlined by nf-core (12). They are intended to be efficient, intuitive for novice users, optimisable for laptop, HPC cluster or the cloud, and scalable from small lab studies to field trials with large populations. A list of individual pipeline processes alongside the default, recommended resource configurations are provided in Supplementary Table S1. Each resource allocation is fully customisable under the Nextflow framework to suit integration under different systems and scheduling software. Dependencies are as simple as installing Nextflow alongside one of either Bioconda (13), Docker (14) or Singularity (15) on a POSIX compatible system, facilitating a high level of flexibility and reproducibility through the use of portable software containers and environments. Each pipeline is fully self-contained and can be easily transferred from one system to another without need for specific, manual installation of the component software. The toolkit is open-source and publicly available on GitHub, allowing users to fork and modify the pipelines at their own discretion including access to the entire change history. The toolkit represents a starting point for the standardisation of DNA methylation profiling in ecological plant epigenetics, and will be actively maintained and expanded upon as additional tools are developed in the future. All pipeline output is streamlined to standard, recognised formats to facilitate interoperability with external software and help create flexible analyses for a wide range of possible experiments, for example when intersecting methylation bedGraph files with gene or transposable element (TE) annotations using BEDTools (16).

## MATERIALS AND METHODS

### Test data

In order to demonstrate selected features from the toolkit, a subset of 23 independent, whole genome bisulfite sequencing libraries (150 bp long paired-end reads) of the deciduous tree species *Populus nigra* were selected from the repository hosted by the European Nucleotide Archive (ENA) at EMBL-EBI under accession number PRJEB44879 (https://www.ebi.ac.uk/ena/browser/view/PRJEB44879). The libraries were sequenced under the broader initiative of the EpiDiverse consortium according to the procedures outlined by Díez Rodríguez *et al.* (manuscript in prep.). This subset represents two clone populations (Supplementary Table S2) derived from cuttings originating from field sites in Germany and Lithuania and cultivated together under common garden conditions. Measurements of leaf flavonol content from the parent generation were derived from observations taken in the field by Díez Rodríguez *et al.* (manuscript in prep.). The reference genome was obtained from the repository hosted by the ENA at EMBL-EBI under accession number PRJEB44889 (https://www.ebi.ac.uk/ena/browser/view/PRJEB44889).

### Whole genome bisulfite sequencing (WGBS)

The EpiDiverse WGBS pipeline derives sequence alignments in BAM format from input NGS reads in FASTQ format and a provided reference genome in FASTA format, which are taken forward to estimate the methylation level over each position under the given methylation context(s), in bedGraph format. The reference genome is optionally indexed by the pipeline itself, or provided alongside the relevant index files to begin with. Mapping of bisulfite sequencing data can be carried out either in ‘high-throughput mode’, with a low memory footprint and a runtime suitable for rapid analysis of population data, or ‘high-sensitivity mode’, with a demonstrable improvement in precision-recall and downstream methylation analysis for non-model plant species, as selected according to corresponding benchmarks (17). Multiple samples can be processed in parallel, and quality control (QC) is performed with a combination of published tools and in-house scripting. Basic visualisation of alignment statistics is performed with samtools stats and the corresponding plot-bamstats tool (18). Methylation values based on coverage are called with MethylDackel (https://github.com/dpryan79/MethylDackel), which also provides QC for M-bias analysis and overlapping paired-end reads.

### Variant calling and sample clustering (SNP)

The EpiDiverse SNP pipeline performs a novel masking procedure which compares individual nucleotides from the bisulfite sequencing alignments, obtained from the EpiDiverse WGBS pipeline, to the reference genome. Joint variant calling is then performed on the masked BAM files, to provide single nucleotide polymorphisms (SNPs) in standard VCF/BCF format which are filtered by the pipeline according to customisable parameters. As SNPs in a cytosine-to-thymine context are obscured in bisulfite data (8), neither variant calling nor sample methylation clustering can be resolved using conventional methods. A simple post-processing procedure for *in silico* manipulation of both base qualities and base nucleotides in bisulfite contexts, following alignment, has been shown to facilitate conventional SNP calling on WGBS data which outperforms equivalent, specialised software (19). This heuristic method has been implemented herein and enables a) downstream analysis with tools that are already well-established for DNA-seq such as Freebayes (20), and b) sample clustering with kWIP (21) which uses k-mer diversity to estimate a distance matrix. Variant calling in this manner can eliminate the need for conventional DNA-seq data alongside bisufite sequencing data, thus reducing sequencing costs for plant ecologists. Basic visualisation of variant statistics is also carried out using bcftools stats and the corresponding plot-vcfstats tool (22).

### Differential methylation (DMR)

The EpiDiverse DMR pipeline analyses statistically significant differential methylation from a collection of sample-specific methylation files in bedGraph format obtained from the EpiDiverse WGBS pipeline, providing the output in a custom BED format. A recent benchmark demonstrated a higher sensitivity for finding DMRs with metilene in comparison to other tools (23). Pairwise comparisons of methylation profiles between groups are therefore made with metilene (24), to derive either differentially methylated regions (DMRs) or positions (DMPs) while also correcting for multiple comparisons. Any annotations in BED format can also be provided by the user, as pre-selected regions for comparison, as an alternative to the default boundary estimation based on the methylation signal. Due to the non-parametric statistical test, each methylation context (CG, CHG, CHH) can be analysed independently (or combined) without any *a priori* assumptions about the underlying distribution of methylation values. Significant DMRs in terms of hyper- and hypo-methylation are visualised using custom Rscripts to provide density plots and heatmaps.

### Epigenome-wide association studies (EWAS)

For a given population of samples, the output derived from previous aspects of the toolkit (i.e. methylation files in bedGraph format, SNP variants in VCF format, annotations such as DMPs/DMRs in BED format) can be combined and processed using the EpiDiverse EWAS pipeline (25) for analysis using the GEM suite (26), in order to study the association between epigenetics, genetics, and environmental metadata through the identification of quantitative trait loci (QTL). These QTLs can be discovered either by taking the full set of methylated positions, in any methylation context, or by first subsetting according to provided annotations (e.g. DMPs/DMRs), or even by taking the provided annotations themselves in place of methylated positions for use as genomic markers, whereby the pipeline will calculate the average methylation level in each case by intersecting the methylated positions. The confounding genetic component can be resolved in each case by providing the SNPs derived in the first place from the same bisulfite data, without the need for conventional whole genome sequencing data alongside.

## RESULTS AND DISCUSSION

The 23 independent WGBS libraries were first mapped in ‘high-throughput mode’ with the EpiDiverse WGBS pipeline, resulting in mapping rates ranging from 78.38% to 80.44% under default parameter settings (Supplementary Table S3). The global methylation level in all contexts is reported in Supplementary Figure S1, alongside a principal component analysis demonstrating the unsupervised grouping of all samples based on the variation in shared methylated sites.

Following alignment, variant calling was performed with the EpiDiverse SNP pipeline to identify SNPs from bisulfite-treated data based on sequence masking and base quality manipulation (19). The total number of variants in each sample are summarised in Supplementary Table S4. Alternatively, the pipeline can attempt to mask short variants and normalise the genetic diversity between samples. As studies on population epigenetics tend to centre around species with low genetic diversity (cf. hierarchical clustering tree on genetic information in Supplementary Figure S2a), a hierarchical clustering based on sequence k-mer diversity (21) after masking short variants can instead give an indication of grouping based on DNA methylation patterns (Supplementary Figure S2b). Such an analysis can facilitate the identification of discrete groups prior to calling differentially methylated positions / regions, without limiting the analysis to only those methylated positions that are shared across all samples by a minimum threshold on sequencing depth. Otherwise, the distance matrix can instead be estimated from the methylation values in the conventional approach following per-sample methylation calling.

**Figure 2.**
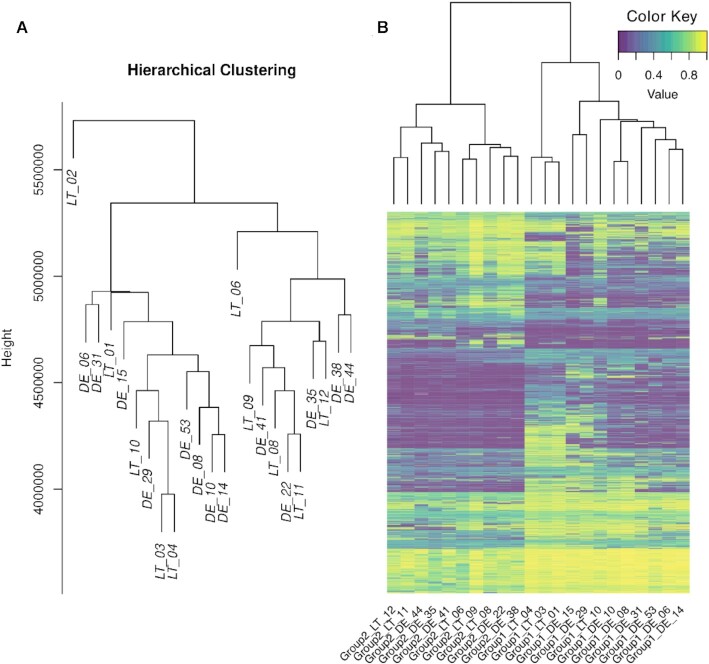
(**A**) Hierarchical clustering of methylated sites (all contexts) derived from the cohort of *P. nigra* samples from populations in Germany and Lithuania, and (**B**) the resulting heatmap of significant DMRs (*q* < 0.05) obtained after cutting the hierarchical tree at 5.25 × 10^6^ to form two discrete groups (leaving LT_02 as outlier). Either plot can be obtained using the EpiDiverse toolkit.

Appropriate groupings of samples are dependent on the specific experimental design of each study. Once identified, they can be subsequently evaluated for differential methylation with the EpiDiverse DMR pipeline, which analyses either all possible pairwise comparisons of groups or each group in relation to a designated control group. Conventionally, groups are identified based on *a priori* knowledge or a global clustering of methylated sites. When grouping in this manner, however, local differences which are perhaps biologically relevant to the study question may be obscured by the global methylation profile and thus not revealed in the subsequent differential methylation analysis. Here, methylated sites (all contexts) obtained from the cohort of German and Lithuanian populations of *P. nigra* were subject to hierarchical clustering and the resulting tree cut at ∼5.25 × 10^6^ to form two discrete groups and one outlier (Figure 2A). The total number of significant DMRs (*q* < 0.05) resulting from the pairwise comparison of these groups are given in Supplementary Table S5, and the corresponding heatmap showing the differential methylation level across the range of selected samples is shown in Figure 2B. Interestingly, the heatmap in some instances shows greater congruency with the clustering based on kWIP in Supplementary Figure S2b (e.g. LT_10, DE_41, DE_44, and a distinct clade with LT_01, LT_03, LT_04), indicating the potential utility as an alternative approach. While still a global clustering analysis, the local information inferred from sequence k-mers may be more robust in identifying groups based on regional differences in comparison to the site-by-site approach.

**Figure 3.**
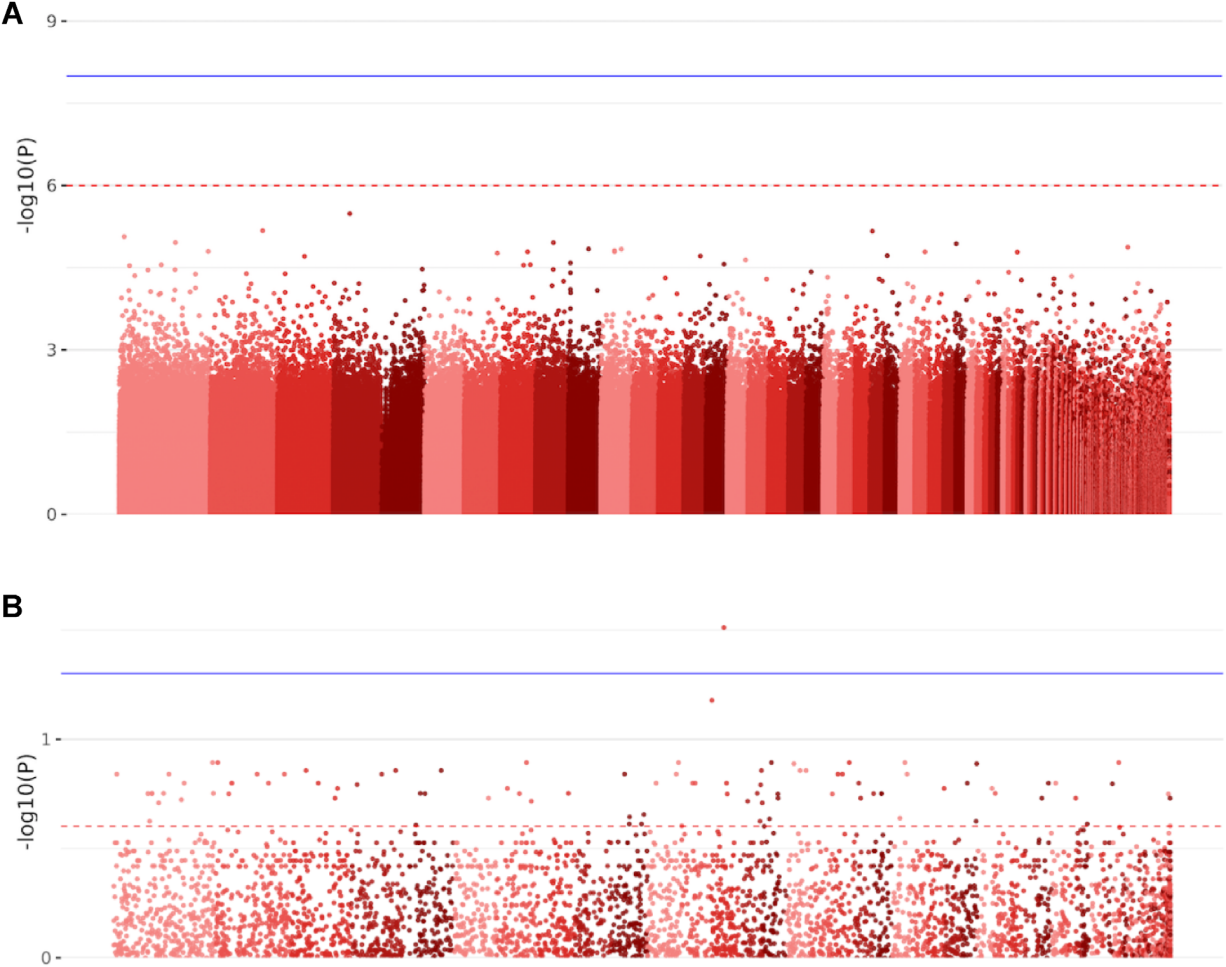
Manhattan plots demonstrating (**A**) the total number of tested positions during EWAS, from the cohort of *P. nigra* samples obtained from populations in Germany and Lithuania, and (**B**) the same analysis performed using significant DMRs instead. At the position-level, none were found to be significant (*P* < 1 × 10^−8^) or even suggestive (*P* < 1 × 10^−6^) based on common thresholds selected to account for the burden of multiple testing. At the region-level it becomes feasible to use Benjamini–Hochberg adjusted *P*-values (*q*), where 92 tests were found below a significance threshold of *q* < 0.25 and one even at *q* < 0.05. The plots are obtained automatically from the EWAS pipeline output (E-model).

Finally, the accumulation of results from the WGBS and DMR pipelines were combined into a small analysis with EpiDiverse EWAS, based on the methylated sites in CG context and subset according to the significant DMRs discovered in the same context, using leaf flavonol content measured in the parent generation as a phenotypic trait. In the case of *P. nigra* the resulting manhattan plot (E-model) in Figure 3 reveals initially no significant QTLs below the common significance threshold of *P* < 1 × 10^−8^, or even below the suggestive significance threshold of *P* < 1 × 10^−6^, based on the global analysis of all methylated sites. The same analysis when conducted however at the region-level revealed a total of 92 significant QTLs (*q* < 0.25) which could be taken forward for further investigation (Supplementary Table S6). A brief inspection of these regions intersected with functional annotations in the *P. nigra* genome returned some features potentially relevant to flavonol content, including genes with homology to *ascorbate-specific transmembrane electron transporter 1*, *caspase family protein* and *mechanosensitive ion channel protein 3* alongside also *methyltransferases PMT2/PMT24*. Regions of hyper- or hypo- methylation may convey a more consistent association among the population of samples and can be more indicative of a mechanism which interacts for example with gene expression. This approach can be therefore more robust than the study of individual methylated sites, depending on the extent of stochastic variation in the DNA methylation signal, but true associations may be missed in regions where DMRs were not identified as a result of local methylation differences which were obscured by global clustering techniques. Furthermore, the incorporation of SNP data into the G-model aspect of the EWAS pipeline can help to resolve any underlying genetic component which may be driving such associations with epigenetic markers.

A typical drawback of any (epi)genome-wide association study is the high burden of multiple testing, necessitating the use of a controlling procedure which can often be excessively conservative due to the high number of negative tests, thus obscuring many genuine biological findings which may be present within the dataset. The common significance threshold of *P* < 1 × 10^−8^ is based on a Bonferroni adjustment limited to a maximum of 1 million tests, regardless of the true number of tests. It is often argued with genetic data that a lack of true sample independence owing to linkage disequilibrium between SNPs can facilitate the use of this more heuristic variant of the Bonferroni adjustment, but statistically speaking this may be less than ideal. A more robust solution would be to reduce the total number of tests in the first place based on *a priori* knowledge. The EWAS pipeline therefore provides a mechanism to subset data based on any such regions provided by the end-user, for example here with DMRs obtained from the DMR pipeline, with the aim to reduce the majority of negative tests while still capturing the majority of positive tests. True positives may still be missed, depending largely on the selection criteria of such regions, though often more can be gained relative to the global analysis of all methylated positions.

## CONCLUSION

The EpiDiverse Toolkit provides a suite of software pipelines for the analysis of ecological plant epigenetics, which adheres to the principles of ‘FAIR’ (Findability, Accessibility, Interoperability, and Reusability). The toolkit combines common procedures, such as mapping and methylation calling, with novel implementations for short variant calling and combining all results within a robust variation of EWAS, with each aspect benchmarked specifically for non-model plant species. This provides a consistent, repeatable framework which not only streamlines computational analyses within-species, but also facilitates more general comparisons between different organisms which may have evolved very different mechanisms involving DNA methylation.

## DATA AVAILABILITY

All pipelines are open-source and publicly available through the https://github.com/EpiDiverse domain. The data used for analysis was generated by the European Training Network “EpiDiverse” to be published in the European Nucleotide Archive, and is otherwise available upon reasonable request to the authors.

## Supplementary Material

lqab106_Supplemental_Files
