# Supplementary material for: EpiDiverse Toolkit: a pipeline suite for the analysis of bisulfite sequencing data in ecological plant epigenetics
Source: NAR Genom Bioinform. 2021 Nov 12;3(4):lqab106. doi: 10.1093/nargab/lqab106 (PMC8598301; doi:10.1093/nargab/lqab106)
Supplement: lqab106_Supplemental_Files [file lqab106_supplemental_files.zip › Supplementary Figures.docx]

| **a)**  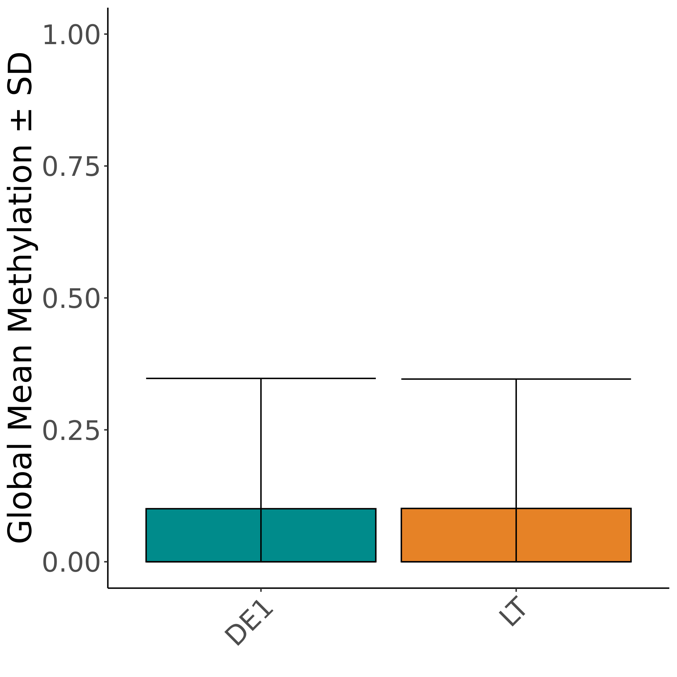 | **b)**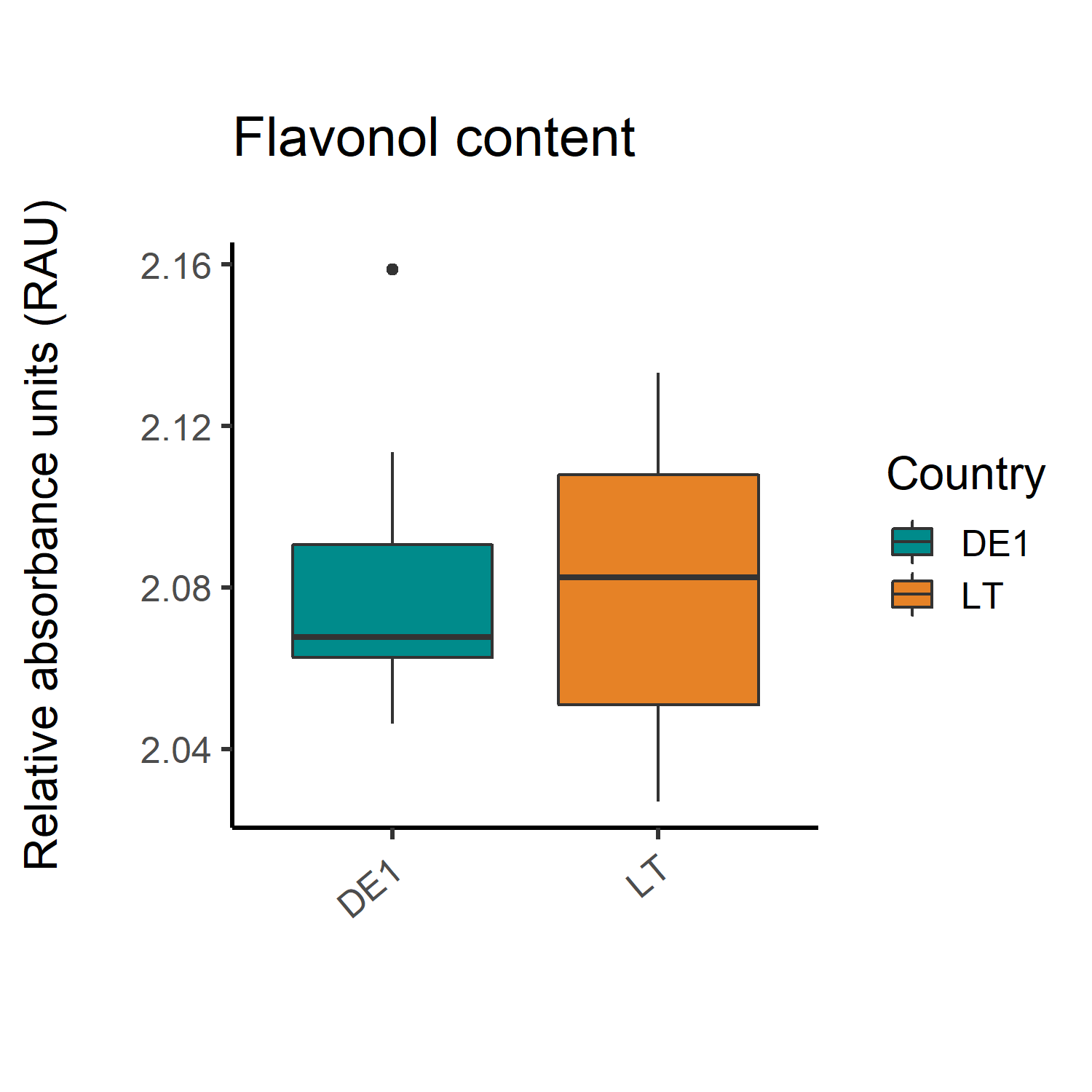 |
| --- | --- |
| **c)**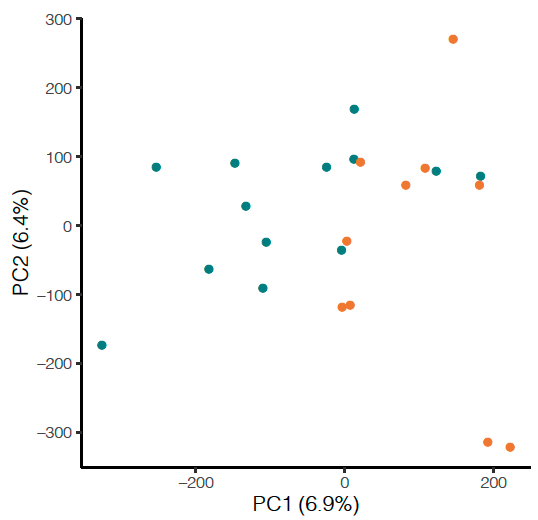 | **d)**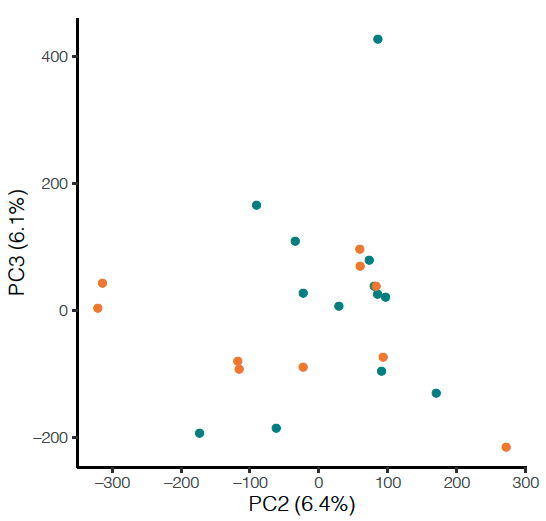 |
| **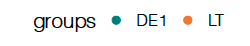** | |
| **Figure S1.** Descriptive statistics of the German (DE1) and Lithuanian (LT) populations of *Populus nigra* samples demonstrating **a)** global methylation levels in all contexts following cultivation under common garden conditions, **b)** leaf flavonol content as measured from samples collected in the field, and Principal Component Analysis (PCA) based on the per-site methylation levels in all contexts between **c)** component 1 and 2, and **d)** component 2 and 3. | |

| 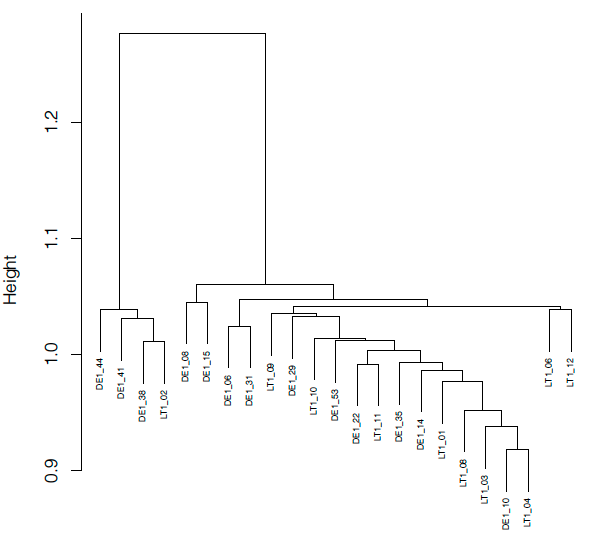**a)** |
| --- |
| **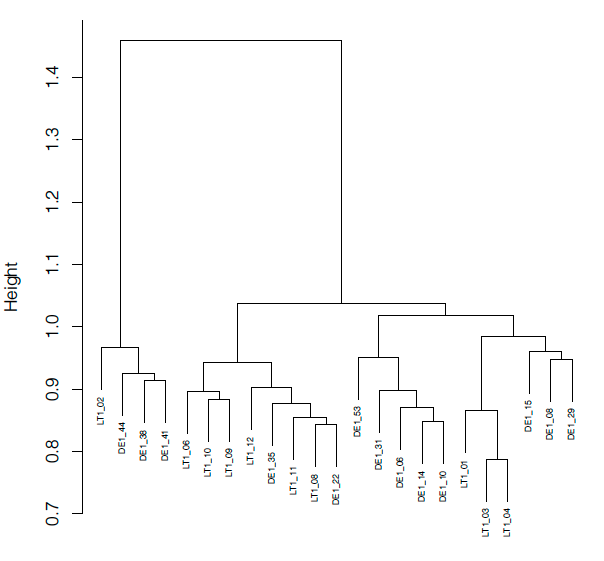b)** |
| **Figure S2.** Hierarchical clustering of mappable FASTQ reads by k-mer diversity, using kWIP, following either **a)** bisulfite masking, or **b)** masking short variants to normalise genetic diversity. |
